# China’s International Trade of Parrots from 1981 to 2022 Based on the CITES Trade Database

**DOI:** 10.3390/ani14213076

**Published:** 2024-10-25

**Authors:** Jinming Zhang, Qingqing Wang, Jianbin Shi

**Affiliations:** School of Environment, Beijing Normal University, Beijing 100875, China; 202221180059@mail.bnu.edu.cn (J.Z.);

**Keywords:** parrot trade, trade control, domestic trade, illegal trade, captive breeding

## Abstract

Parrots are popular with pet consumers and are one of the most traded groups of live birds around the world. China has been considered as an important country in bird trades, but we know little about China’s import and export of parrots. Using the latest data available from the CITES Trade Database, we analyzed China’s import and export of CITES-listed live parrots from 1981–2022 to better understand the patterns and dynamics of these trades. China imported 155,339 parrots of 173 species and exported 608,987 parrots of 42 species during the study period with an annual average of about 18,500 individuals, accounting for c. 5% of the global average number. The most imported parrots to China were Grey Parrot (*Psittacus erithacus*) and Monk Parakeet (*Myiopsitta monachus*), while the most exported ones were Fisher’s Lovebirds (*Agapornis fischeri*), Rosy-faced Lovebirds (*Agapornis roseicollis*), and Yellow-collared Lovebird (*Agapornis personatus*). Since the early 21st century, China’s imports of parrots gradually increased, but exports decreased more pronouncedly, making China’s international parrot trade volume declining in the past 20 years. China’s international parrot trade routes also changed over time, with the destination of parrot exports shifting from Europe to Africa and the Arabian region, while more parrots being gradually imported from countries in South America and Africa. The vast majority of parrots exported from China were captive-bred non-native species like Fisher’s Lovebirds, while a substantial proportion of imported parrots were wild-sourced and the proportion has been increasing in the last decade. The fact that China exported much more parrots than imported suggests existence of large-scale parrot farming and domestic parrot trade in China. Attention needs to be paid to monitoring China’s import of increasing proportion of wild-sourced parrots from South America countries and its potential impacts on their wild populations. Close monitoring of and more studies on domestic and illegal parrot trade are essential to fully understand the role China plays in the international trade of parrots.

## Simple Summary

Using data from the CITES Trade Database, we analyzed China’s imports and exports of CITES-listed parrots from 1981 to 2022 to better understand the characteristics of these trades. China imported 155,339 parrots of 173 species and exported 608,987 parrots of 42 species with a yearly average of about 18,500 individuals, accounting for c. 5% of the global average number. The most imported parrots were Grey Parrot (*Psittacus erithacus*) and Monk Parakeet (*Myiopsitta monachus*), while the most exported were Fisher’s Lovebirds (*Agapornis fischeri*), Rosy-faced Lovebirds (*Agapornis roseicollis*), and Yellow-collared Lovebird (*Agapornis personatus*). China’s imports of parrots gradually increased, but exports decreased more pronouncedly, making China’s international parrot trade volume declining in the past two decades. The major destination of parrots exported from China shifted from Europe to Africa and the Arabian region, while more parrots were gradually imported from South America and Africa. The vast majority of parrots exported from China were captive-bred, while a substantial and increasing proportion of imported parrots were wild-sourced. Attention needs to be paid to monitor China’s import of increasing proportion of wild-sourced parrots and its potential impacts on their wild populations.

## 1. Introduction

Parrots (Psittaciformes), comprising about 400 species, are widely distributed across continents and oceanic islands [1], and have been popular with consumers as caged birds and companion pets throughout human history around the globe, possibly because of their diverse plumage colors, multiple sizes, and ability to imitate human sounds [2]. There is a long history of trading and keeping parrots as pets in many countries and cultures, and parrots are among the most traded of all birds in the world [3]. A recent study shows that Psittaciformes make up about 90% of the live bird trade monitored by the Convention on International Trade in Endangered Species of Wild Fauna and Flora (“CITES”) [4], with Psittacidae being one of the twenty most traded families in the global bird trade [5]. According to CITES data, over 19 million individual live parrots (with an annual mean of almost half million) of 336 species were legally traded among countries from 1975 (when CITES entered in force) to 2015 [6].

Several threats to parrots, such as habitat loss and degradation, hunting and trapping, and wildlife trade, have rendered parrots one of the most threatened and endangered taxa of birds. Parrots stand out among birds for their poor conservation status [7,8,9,10]. According to IUCN Red List [11], almost 60% of all parrot species are experiencing global population declines, with 98 species (25.7%) being considered globally threatened with extinction and 51 species near threatened (13.4%), 20 of them being critically endangered and 27 endangered. For example, the wild populations of the African Grey Parrot (*Psittacus erithacus*) have shrunk by over 50% in many areas due to overharvesting and forest loss [12]. All species of parrots in the order Psittaciformes, except for four species of parrots (i.e., Rosy-faced lovebird *Agapornis roseicollis*, Budgerigar *Melopsittacus undulatus*, Cockatiel *Nymphicus hollandicus*, and Rose-ringed parakeet *Psittacula krameri*), are listed in CITES’s appendices [13].

Trade of wild parrots in large numbers could not only cause a decline in wild parrot populations, but could further affect the ecosystem services they provide [14,15]. Without effective management, overexploitation can lead to the collapse of wild populations of some parrots, resulting in extinction and the loss of their ecosystem services [15]. Additionally, unsupervised international trade of parrots may well lead to species invasion and the spread of infectious diseases that may pose threat to biodiversity, economy and human health in importing countries/regions [16,17,18,19,20,21]. For example, Monk Parakeet (*Myiopsitta monachus*), native to some South America countries, has spread and become highly invasive with international trade in many other countries and regions [6,22]. The beak and feather disease virus (BFDV) has been recently found to be transmitted to eight countries possibly as a result of the global parrot trade [18,19]. It is thus important to better understand the international parrot trade involving China to develop effective measures protecting biodiversity from depletion and preventing the invasion of alien species as well as the spread of potential diseases within China.

Some studies have been conducted on international trade in parrots based on the CITES Trade Database, focusing on changes in their trade profiles and the influencing factors. The trends in international trade and ecological status of CITES-listed live Australian native parrots have been influenced by various political and economic factors [23]. The changes in trade volumes and trade paths of African Grey Parrots and Timneh Grey Parrots (*P. timneh*) since 1975 have been heavily influenced by the CITES trade review in the early 1990s, the implementation of the Wild Bird Conservation Act (WBCA) in the US starting in 1992, the European Wild Bird Trade Ban (EU ban hereafter) in 2005, and the avian influenza pandemic outbreak starting around 2000 [24]. These events, together with rapid population growth, increasing affluence, expanding international travel routes, the diffuse use of Internet technology, and cultural shifts in Asia, may have contributed to the gradual change in the international parrot trade pattern, with Asia replacing North America and Europe as the new parrot trade hotspot [3,10,21,25,26,27]. A more recent study reveals the spatial–temporal changes in trade volumes and sources of parrots, the topology of the trade network, and the factors influencing the global parrot trade between 1975 and 2016 [10].

China is rich in wild bird resources with over 1400 bird species [28]; meanwhile, it is also one of the major countries involved in international and domestic bird trade (and more broadly wildlife trade) with long history of bird keeping [29,30]. A large number of birds have been traded in China to meet the demand for birds for various purposes, although it is hard or even impossible to assess the status of domestic bird trade in China. There have been sporadic surveys and studies of domestic bird markets in China [29,31,32], suggesting domestic bird trade is common, particularly before COVID-19. Eighteen surveys of a single bird market in Haerbin City of China from November 2016 to November 2017 recorded 18,729 individual birds of 117 species, among which 11,662 birds of 108 species were wild-caught [32].

Among the over 1400 bird species in China, only 9 parrot species are native to China, and all these species have been listed as the second-class State key protected wild animals (II), with 6 species being listed as near-threatened and 3 others as least concerned on the IUCN Red List [11]. Parrots remained the most traded group of China’s international trade in CITES-listed birds during the periods of 1981–2010 [27] and 2010–2019 [33]. A previous study demonstrates that China imported more than 90,000 live birds of c. 130 species and exported 603 live birds of 10 species from 2010 to 2019, with 86% of the imported and 75% of the exported birds being parrots [33]. Parrots are also the most traded group of birds observed in the domestic bird markets [34]. There seems to be an increase in online trade in parrots in China as indicated by a study which counted a total of 5862 individuals of 46 parrot species during four surveys of Taobao platform (equivalent to eBay) [35].

Several recent studies based on the CITES Trade Database have focused either on parrot species of high conservation concerns like the African Grey Parrot and Timneh Parrot [24,36] or on the analysis of important trade hubs like Singapore [37] or on countries with endemic parrots like Australia [38], but no recent studies have been conducted specifically on the import and export of parrots by China. China’s import and export of parrots are supposed to be multifaceted and unique because China is rich in bird species but lacks parrot species. China is also a globally significant consumer market for parrots, and legal and illegal parrot trades co-exist. Furthermore, there has been expectation of growing demand for parrots as companion pets, particularly for children and old people in China [30]. However, we do not know yet the role China plays in international parrot trade, and it is unclear what parrot species are and their respective abundances that are being traded with China. Such information is important for developing proper policies ensuring conservation and sustainable use of parrots. The study of China’s import and export of parrots can also contribute to understanding the domestic bird markets and even illegal bird trade in China. We thus used the CITES Trade Database to analyze the patterns and trends of China’s import and export of CITES-listed live parrots from 1981 to 2022, focusing on identifying the main parrot species involved, the trade volume, trade partners, geographic scope, and sources. We paid special attention to the possible effects of the WBCA (1992), the outbreak of avian influenza around 2000, and the 2005 EU ban on the dynamics of China’s import and export of parrots because studies have shown they have influenced global trades in parrots and other live birds in other countries or globally [10,21,27,39]. Our findings may provide a basis for better management of China’s international trade in parrots, for determining the possible impacts of international parrot trade on their wild populations, and for assessment and early warning of potential bio-invasion risks to be caused by international trade of parrots in China [40].

## 2. Materials and Methods

### 2.1. CITES Trade Database

The Convention on International Trade in Endangered Species of Wild Fauna and Flora (CITES) requires the Parties to regulate the international trade of endangered species in order to avoid undesirable consequences such as possible extinction of species due to improper trade on a global scale. The CITES Trade Database (available online: https://trade.cites.org) is the main tool for monitoring the implementation of CITES and the level of international trade in animal and plant species listed in the CITES Appendices. Detailed information on the source, quantity, and purpose of trade in specimens is provided by the parties in their annual reports, as defined in the Guidelines for the preparation and submission of annual reports (CITES Notification to the Parties No. 2023/039 Annex 1). The data in the database are derived from the annual reports of import and export trades submitted by the Parties to the CITES Convention before October 31 of each year. Due to the timing of annual report submission, statistics on the full trade of a species are generally available two years after its international trade has occurred [41]. Currently, the CITES Trade Database holds over 25 million trade records for more than 40,000 CITES-listed wildlife species.

Discrepancies in the volume of trade reported by importers and exporters can occur due to several reasons, for instance, the trade not reported by the importing Party, year-end trade (when export permits are issued at the end of a given year but not received or reported by the importers until the following year), or inconsistencies in the way Parties record and report transactions (e.g., using different terms or units for the same items in trade). This is closely related to the level of development, trade management systems, and species protection policies of each Party. Despite these possible discrepancies, the CITES Trade Database remains the most comprehensive long-term database available for international trade in wildlife and has been proven to be important for determining species trade patterns and exploring temporal and geographic trends in wildlife trade [23,24,38,42,43]. For example, it may be impossible to determine whether there are intentional mislabeling or labeling wild-sourced specimens as captive-bred ones in the trade records using only the CITES Trade Database, but the Parties could detect such problems by increasing the sampling rate [44].

### 2.2. Data Acquisition and Analyses

We downloaded the China’s parrot import and export trade data between 1981 and 2022 (the latest available data are for 2022) from the CITES Trade Database on August 6, 2024 in the form of a comparative tabulation report, which presents the data in an aggregated format and provides exporter- and importer-reported quantities separately. We then extracted the import and export trade records classified as “live” and “Psittaciformes” from the report. For each transaction, we collected the following information: (1) species classification; (2) year; (3) exporter and importer; (4) trade volume (number of individuals); (5) source of traded objects; and (6) trade purpose.

We included all records of trade in the order Psittaciformes regardless of whether the trade was reported at lower taxonomic levels (e.g., records of taxon Psittaciformes spp.) to count the trade volume [41]. In some cases where the number reported by the importer and exporter differed for a given record, the higher number was used as the trade volume for that record [21,38].

The scientific names of the parrots in the CITES Trade Database and in our study follow CITES standard nomenclature (as outlined in Resolution Conf. 12.11 (Rev. CoP18)). CITES standard nomenclature does not recognize the split of *Psittacus* parrots into two distinct species (i.e., *P. erithacus* and *P. timneh*), so all records related to the genus *Psittacus* were downloaded, and no attempt was made in this study to distinguish trade in Timneh Parrots and Grey Parrots.

Because not all parrot species are on the CITES Appendices and data reporting for these species are not consistent, our study also faced the problem of incomplete information in some records. This study found that some transaction records involving China’s import and export trade of parrots lacked information on species classification or source of trade/trade purposes, but most of these occurred before the early 1990s and became rare gradually in the later years.

## 3. Results

### 3.1. Species and Number of Parrots China Imported and Exported

During the study period, China imported 155,339 live parrots of 173 species, and most of them were from Psittacidae (93%).

In terms of the number of individuals, the major parrot species China imported were Grey Parrot, Monk Parakeet, Yellow-collared Lovebird (*Agapornis personatus*), Sun Conure (*Aratinga solstitialis*), and Rose-faced Lovebird (*Agapornis roseicollis*). A total of 14 out of the top 15 imported parrot species were from Psittacidae, except the Coconut Lorikeet (*Trichoglossus haematodus*). The top 15 imported parrot species accounted for 74% of the total imported (Table 1).

China exported 608,987 live parrots of 42 species during the same period, and the vast majority of them belonged to Psittacidae (99.7%). The exports of parrots from China were dominated by three lovebird species, i.e., Fischer’s Lovebird (*Agapornis fischeri*), Rosy-faced Lovebird and Yellow-collared Lovebird, and they accounted for 98.7% of the total exported parrots. The number of individuals from the top 15 exported species accounted for 99.5% of the total exported (Table 1).

**Figure 1:**
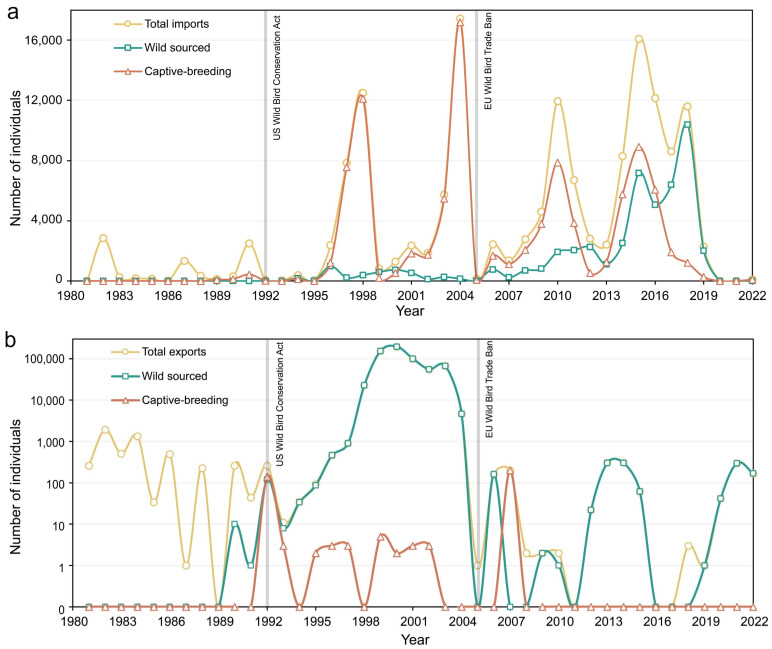
Variation in the number of individual parrots China imported and exported from 1981 to 2022 ((**a**): Imported; (**b**): Exported).

Between 1981 and 2022, China exported far more individual parrots than it imported, but both imports and exports displayed pronounced interannual variation (Figure 1). China’s import of parrots fluctuated and reached its first peak in 1998 (n = 12,504), after which the import quantity decreased sharply in 1999 and recovered gradually to arrive at its second (and also the highest) peak in 2004 (n = 17,433). However, the import quantity dropped sharply to only 162 in 2005. Since 2005, the import quantity fluctuated but with a trend of steady increase and reached its third peak in 2015 (n = 16,093). The import trade experienced a decline in the period from 2016 to 2022 with the import quantity in 2020 and 2021 being zero (Figure 1a).

The export quantity between 1981 and 2022 displayed a little different trend from import quantity (Figure 1b). Prior to 1998, China’s export of parrots fluctuated between a few and 2000 per year, with no export records for some years (e.g., 1989). It increased rapidly thereafter, reaching a peak in 2000 (n = 196,065), after which the number of parrots exported from China declined until the lowest value (only one) in 2005. Although exports slowly rebounded in subsequent years, they never reached the export levels recorded in 2000 again, fluctuating between 0 and 308 individuals per year until 2022. In particular, during the last two decades (2005–2022), China exported only 443 individuals, and there were no export records in the years of 2011, 2016, and 2017 (Figure 1b).

**Figure 2:**
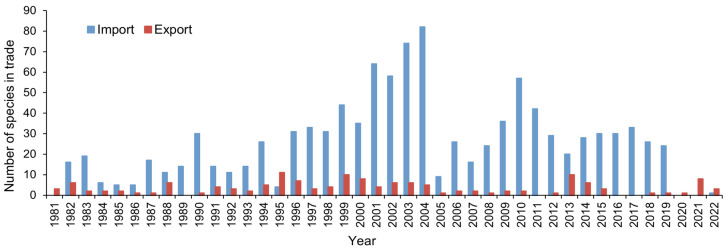
Number of parrot species China imported and exported from 1981 to 2022.

China exported much more individual parrots than it imported, but it imported more parrot species than it exported in each year, except 1995 (Figure 2). Compared to the imported parrot species, the number of exported parrot species was more stable between years and usually remained below 10 species with small fluctuation (Figure 2).

### 3.2. Sources and Destination of Traded Parrots

**Figure 3:**
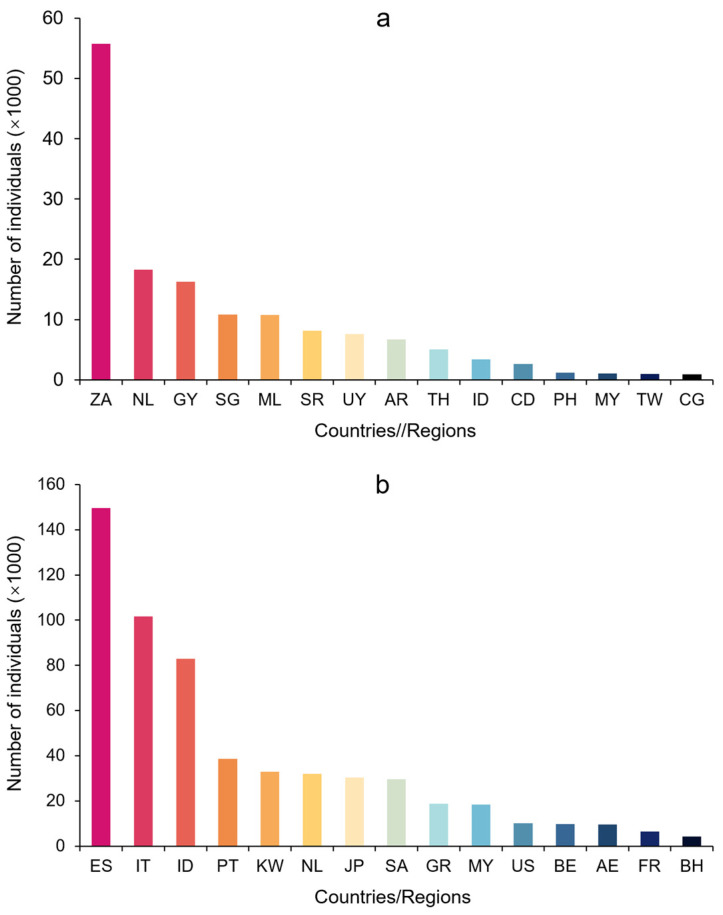
The top 15 countries/regions involved in parrot trade with China from 1981 to 2022 ((**a**): Source countries/regions of parrots imported to China; (**b**): Destination of parrots exported from China). The origin (**a**) and destination (**b**) include the following countries/regions: AE—United Arab Emirates; AR—Argentina; BE—Belgium; BH—Bahrain; CD—Congo (RDC); CG—Congo (Brazzaville); ES—Spain; FR—France; GR—Greece; GY—Guyana; ID—Indonesia; IT—Italy; JP—Japan; KW—Kuwait; ML—Mali; MY—Malaysia; NL—Netherlands; PH—Philippines; PT—Portugal; SA—Saudi Arabia; SG—Singapore; SR—Suriname; TH—Thailand; TW—Taiwan, China; US—United States; UY—Uruguay; ZA—South Africa.

**Figure 4:**
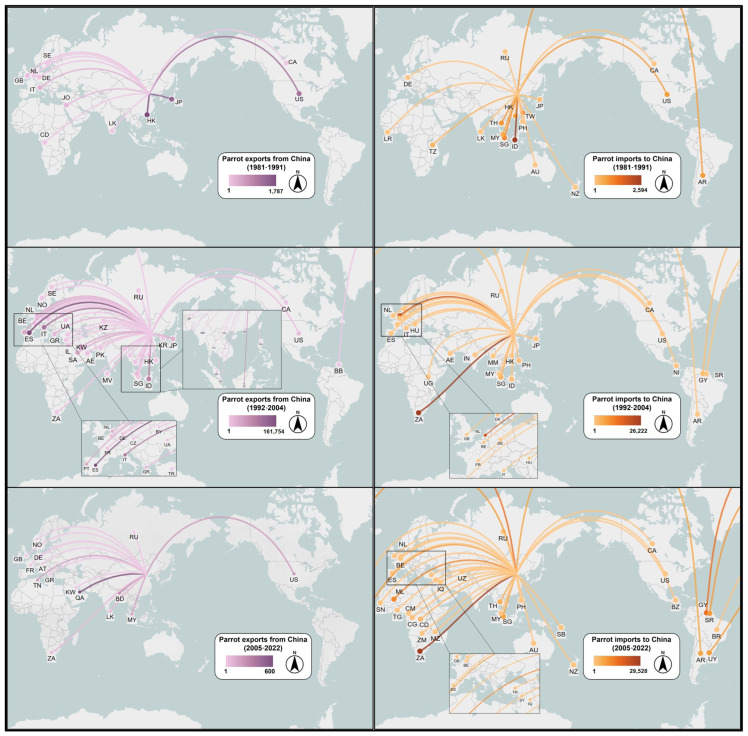
Temporal parrot trade routes of China from 1981 to 2022. Countries/regions: AE—United Arab Emirates; AR—Argentina; AT—Republic of Austria; AU—Australia; BB—Barbados; BD—Bangladesh; BE—Belgium; BH—Bhutan; BR—Brazil; BY—Belarus; BZ—Belize; CA—Canada; CD—Congo (RDC); CG—Congo; CM—Cameroon; CY—Cyprus; CZ—Czech Republic; DE—Germany; DK—Denmark; ES—Spain; FR—France; GB—United Kingdom of Great Britain and Northern Ireland; GR—Greece; GY—Guyana; HK—Hong Kong, China; HU—Hungary; ID—Indonesia; IL—Israel; IN—India; IQ—Iraq; IT—Italy; JO—Jordan; JP—Japan; KR—Republic of Korea; KW—Kuwait; KZ—Kazakhstan; LK—Sri Lanka; LR—Liberia; ML—Mali; MM—Myanmar; MV—Maldives; MY—Malaysia; MZ—Mozambique; NI—Nicaragua; NL—Netherlands; NO—Norway; NZ—New Zealand; PH—Philippines; PK—Pakistan; PT—Portugal; QA—Qatar; RU—Russian Federation; SA—Saudi Arabia; SB—Solomon Islands; SE—Sweden; SG—Singapore; SN—Senegal; SR—Suriname; SY—Syrian Arab Republic; TG—Togo; TH—Thailand; TN—Tunisia; TR—Türkiye; TW—Taiwan, China; TZ—United Republic of Tanzania; UA—Ukraine; UG—Uzbekistan; US—United States; UY—Uruguay; UZ—Uzbekistan; VN—Viet Nam; ZA—South Africa; ZM—Zambia.

Between 1981 and 2022, 53 countries or regions exported parrots to China. Most parrots imported by China came from South Africa and Mali in Africa, the Netherlands in Europe, Guyana, Uruguay, and Argentina in South America, and Singapore, Thailand, and Indonesia in Southeast Asia (Figure 3a and Figure 4). The top 15 countries or regions exported a total of 149,567 parrots to China, accounting for 96.3% of the total imported parrots. Although South Africa, a major parrot exporter, started to export parrots to China only in the late 1990s, the number of parrots South Africa exported to China alone accounted for 38.7% of the total parrots imported to China.

After 2010, European countries were no longer the main source of parrot imports to China, while South Africa and Mali in Africa and Guyana, Suriname, Uruguay, and Argentina in South America together exported over 60,000 parrots to China, accounting for 84.5% of the total parrot imports to China between 2011 and 2022 (Figure 3a and Figure 4).

China exported parrots to 46 countries or regions (e.g., Hong Kong and Taiwan of China), with the main destinations being Europe (Spain, Italy, Portugal, the Netherlands, etc.), Southeast Asia (Indonesia, Japan), and the Middle East (United Arab Emirates, Qatar) (Figure 3b and Figure 4). Two European countries, Spain and Italy, imported 251,148 live parrots from China, accounting for 41% of China’s total exports of parrots. The top 15 countries or regions importing parrots from China received approximately 574,850 parrots, accounting for 95% of the total parrots exported from China.

The number of parrots China exported to major European countries (Spain, Italy, Portugal, the Netherlands, Greece, Belgium, and France) varied considerably over time (Figure 4). From 1981 to 1992, China exported only 303 parrots to European countries (Italy and the Netherlands), while in the following decade, from 1993 to 2004 (before the EU ban came into effect in 2005), China exported 368,425 parrots to the seven European countries, accounting for 64% of the total exports of parrots. After 2005, China exported 1581 parrots with only two records of export of one individual each to France and Greece in 2013. Instead, China mainly exported parrots to the Arabian Peninsula countries (e.g., Qatar, n = 600), Bangladesh (n = 300), and the United States (n = 205), although the number remained relatively low (Figure 3b).

From 1981 to 2022, China exported 10,117 parrots to the United States, among which 1236 were exported from 1981 to 1992, while 8881 were exported after 1992 when the US WBCA was introduced. The majority of the parrots exported from China to the US were captive-bred Fischer’s Lovebird (8200), Rosy-faced Lovebird (250), and Yellow-collared Lovebird (200). Although 206 parrots exported to the US were labelled as wild-sourced, they were re-exported from China: 150 Orange-winged amazons (*Amazona amazonica*) and 50 Red-and-green Macaws (*Ara chloropterus*) originating from Guyana.

### 3.3. Purposes and Sources of Traded Live Parrots

About 86% of the total parrots imported to China were for commercial purposes, while 6% and 4% of the imported parrots were for captive breeding and zoo display purpose, respectively (Table 2). Comparatively, China exported parrots for a more specific purpose, with 99.8% of the exported parrots being for commercial purposes (Table 2).

The vast majority of parrots exported from China originated from captive breeding (99.0%), while only about 63% of imported parrots were from captive environments (C, F) and 31% of imported parrots were wild-caught (W) (Table 3; Figure 1), and the proportion of wild-caught sources tended to increase gradually over the last decade. Since 2010, nearly half (49%) of China’s imported parrots originated from wild-caught sources, compared to only 9% prior to 2010. By contrast, about 99% of parrots exported from China, either before or after 2010, were captive-bred.

### 3.4. China’s Import and Export of Grey Parrots

China imported 21,201 Grey Parrots from 1981 to 2022 mainly for commercial use (19,390, 92% of the total) and captive breeding (610), with 99% of the imports (20,994) occurring in 2016 and before. After the Grey Parrot was upgraded to CITES Appendix I in 2016, China imported only 200 Grey Parrots (as Appendix II) in 2017 for commerce and 4 (as Appendix I) between 2017 and 2019 for personal use.

Although 200 Grey Parrots imported by China in 2017 were labeled as wild-caught in origin, they were imported from Singapore with original source from the Democratic Republic of the Congo (Congo (K)) (Table 4) and were imported to China as Appendix II species.

The Grey Parrots imported by China mainly came from South Africa, Mali, Singapore, Congo (K), the Republic of the Congo (Congo (B)), and Mozambique, especially from South Africa which exported 10,787 (51% of the total) Grey Parrots to China. About one-third of the Grey Parrots imported to China were labeled as wild-caught, but because some countries (e.g., South Africa, Singapore, and the United Kingdom) were not the origin country of Grey Parrots, those Grey Parrots that were exported from these countries and labelled as wild-caught were actually re-exported from these countries to China (Table 4).

From 1981 to 2022, China exported 241 Grey Parrots in and before 2014, mainly to Singapore (120) and Greece (100), and only one in 2019. In total, 7 out of these 242 Grey Parrots were labelled as wild-caught, but most of them originated in Nigeria (n = 6).

### 3.5. China’s Export of Its Native Parrots

Four out of the nine parrot species native to China were exported from China in the study period: 212 Rose-ringed Parakeets (*Psittacula krameri*) (Appendix III), 93 Red-breasted Parakeets (*Psittacula alexandri*) (Appendix II) with 80 individuals from the wild; 3412 Lord Derby’s Parakeets (*Psittacula derbiana*) (Appendix II) with 67 from the wild before 1993; and 1 Alexandrine Parakeet (*Psittacula eupatria*) (Appendix II). The vast majority of these exports occurred prior to 1995.

Of all the parrots exported from China, 364 parrots were labelled as wild-caught, among which 147 (4%) were wild-caught native Red-breasted Parakeet (80) and Lord Derby’s Parakeet (67). The others, although labelled as wild-caught, were re-exported from China, because none of them originated in China, such as the Grey Parrot (7), the Orange-winged Amazon (150), and the Red-and-green Macaw (50).

## 4. Discussion

This study found that China imported 155,339 parrots of 173 species and exported 607,986 parrots of 42 species, totaling approximately 760,000 parrots from 1981 to 2022 with an annual mean of about 18,500. There are no accurate estimates of international trade of parrots in the world since CITES entered into force, but two studies provide such an estimate. One study estimates that more than 16 million parrots of 321 species were involved in international trade between 1975 and 2016 with a yearly average of about 400,000 [10], and another study estimates over 19 million parrots of 336 species between 1975 and 2015 with an annual mean of about 500,000 [6]. The yearly average number of parrots China imported and exported from 1981 to 2022 accounts for about 5% of the global average between 1975 and 2016.

Although there was significant interannual variation in China’s imports and exports of parrots, the temporal trends of the import and export differed. The import trade generally showed an increasing trend, while the export showed a decreasing trend, especially during the past two decades (2005–2022) when China exported 443 parrots only. Several factors may have contributed to China’s declining export of parrots. For example, China enacted a policy in 1999 that prohibits capturing, selling, or exporting wild-caught birds except for scientific purposes [27]. The EU ban on import of wild-caught birds from 2005 may also partially explain why China’s export of parrots has continued to decline and remain relatively low since 2005.

The diversity of parrot species imported to China was much higher than that of the exported ones, and the major imported and exported parrot species differed. The most imported parrots to China were Grey Parrots, followed by Monk Parakeets, Sun Parakeets, and Orange-winged Amazons, which are generally larger, rarer, and more expensive [35]. Meanwhile, the most exported parrots from China were several lovebird species which are generally smaller, easier to breed and keep, and relatively cheaper [35], and almost all of the exported parrots were captive-bred. More species of parrots were imported possibly to meet China’s pet consumers’ pursuit for diverse ornamental values and other characteristics (like ability to mimic human speech) of the parrots. China’s State Forestry Administration (SFA) issued a list of terrestrial wildlife with mature domestication and breeding techniques and large wild populations for legal captive breeding, including Fisher’s Lovebirds, Rosy-faced Lovebirds, and Yellow-collared Lovebirds, Cockatiels (*Nymphicus hollandicus*), Budgerigars (*Melopsittacus undulatus*), etc., in August 2003 (available online: http://law.foodmate.net/show-200800.html (accessed on 20 September 2024)). As a result, a large quantity of several lovebirds was imported to China as ornamental pets for mass captive breeding, and some of these parrots eventually became the major parrot species exported from China.

However, this trend of importing a large number of lovebirds has changed over time. In the past 20 years or so, Monk Parakeet originating from some South American countries has replaced lovebirds as the major imported species to China. China has shifted to importing live parrots from South Africa, Mali, Guyana, Surinam, Uruguay, and Argentina, and the proportion of wild-sourced parrots has increased, while the major destination of exported parrots has shifted to the Arabian region. In fact, a significant proportion of parrots imported to China during the study period has been wild-caught from South American countries such as Guyana, Surinam, Uruguay, and Argentina.

Our study revealed that China has imported an increasing proportion of wild-caught parrots and nearly 50% of the imported parrots originated from wild-caught sources since 2010, most of them from South America and Africa countries. This warrants close monitoring of such importation and analysis of its potential impact on their wild populations.

The outbreak of highly pathogenic avian influenza H5N1 in Asia and China in the early 2004 might have contributed to the sharp reduction in China’s export of parrots around 2005. Immediately after the outbreak, over 40 countries/regions banned temporarily imports of poultry and poultry products from China, and China strengthened import and export inspection and quarantine [45], which might have negatively influenced the international trades of live birds, including parrots, with China. The outbreak of H5N1 might have mainly generated immediate and short-term effects, while the implementation of the EU ban, adopted in 2005 first as a temporal measure to prevent the spread of avian flu and other diseases, and since 2007 as an indefinite measure also focused on conservation and animal welfare [17], might have had long-term and lagging effects on the international trade of live birds. In fact, the number of parrots exported from China already began to decline sharply in 2004, possibly attributable more to the impact of avian influenza outbreak in early 2004, while the reduction in parrot exports to one individual in 2005 was largely due to the combined effect of these two factors. The fact that the number of parrots exported from China continued to remain low after 2005 seems to be more largely due to the long-term impact of the EU ban, and European countries are no longer the main destination of China’s exported parrots since 2005.

Although some studies have indicated that the US’s WBCA (1992) has had a significant impact on global trade in wild birds including parrots, this regulation seems to have had limited impact on China’s import and export of parrots [21,27,33,46]. There was no substantial change in import and export trade of parrots between China and the US before and after the introduction of the US’s WBCA, and even after 1992, there were still relatively frequent trades of parrots between the US and China, so that, relatively speaking, the US replaced EU as one of the important destinations of China’s parrot exports after 2005, although the total number of parrots exported to the US was very small (only 218). This may be related to the fact that neither China nor the US are major producers of native parrots, and the parrots involved in the China–US trade are almost all captive-bred.

Although the Grey Parrot is the most imported parrot species to China, the majority of its imports occurred prior to 2015 and most of them were from captive breeding sources. After the Grey Parrot was upgraded to CITES Appendix I in 2016, records show that China imported 204 Grey Parrots in 2017 for commercial use, among which 200 individuals were re-imported from Singapore with original source from Congo (K) and were imported as Appendix II species. It may be reasonable to suspect that this transaction actually took place in 2016 or even in 2015 when the Grey Parrot could still be legally traded globally as a CITES Appendix II species, but was recorded as in 2017 mistakenly in the CITES Trade Database.

More captive-bred parrots are involved in global trade than wild-sourced ones [47,48]. Similar to this, China exported far more parrots than it imported, and the vast majority of exported parrots were captive-bred ones that are not native to China, which suggests the existence of a large-scale parrot farming and domestic parrot trade in China. As an example, it is estimated that over one million Fischer’s Lovebirds and other lovebirds are in stock legally in Shangqiu City of Henan Province, and the history of captive breeding these lovebirds has lasted for over 30 years there (available online: https://www.chinairn.com/hyzx/20211111/180550239.shtml (accessed on 12 December 2022)) [49].

There have been no detailed studies on domestic parrot trade in China, and it is hard or even impossible to assess the status of domestic parrot trade in the country. However, the nation-wide survey of over 200 bird markets in 2016 and 2017 [34] and the online surveys of Taobao e-trading platform from 2015 to 2016 [35] may suggest domestic parrot trade be common across the country with a large volume of traded individuals. For example, a market survey in 2016–2017 [34] found 30.63% of the total birds observed for sale were parrots (140,723) of 43 species, while a study of 12 typical bird markets in China in 2021 and 2022 revealed that over 60% of the total observed individual birds for sale were captive-bred parrots from Psittaciformes (authors´ own unpublished data). Similar to the situation in many other countries or regions (e.g., Japan [46]), the parrot trade dominates both legal and illegal bird trade markets in China in recent years [27,33]. Meanwhile, illegal hunting and trade of wildlife (including birds and parrots) represent a serious threat to wildlife, although Chinese government has undertaken great efforts to deal with them [50,51].

Therefore, more monitoring and intervention efforts are needed to prevent captive breeding from being used as a cover for the wild-sourced trade (“laundering”), meanwhile wildlife enforcement needs to be strengthened to crack down on illegal parrot (wildlife) trade. One way to prevent laundering is using certification and labeling systems [10]. For example, as a pilot, China currently requires parrot farmers to use individual identification systems (e.g., leg rings and matching ID cards) to label and track each individual of the four parrot species which are allowed to keep in captivity for breeding and domestic trade, i.e., Fisher’s Lovebird, Purple-bellied Lory (*Lorius hypoinochrous*), Green-cheeked Parakeet (*Pyrrhura molinae*), and Monk Parakeet [52].

Four species of parrots native to China were recorded for export from China, but their trade volume was not high, the proportion of wild-sourced was small, and most of the export trade occurred before 2000. No study has been conducted to assess the effects of the export of these parrots on their wild populations in China, so there is a need to monitor the trade (both international and domestic) of these parrots and to assess their potential impact.

International trade is recognized as an important and rapidly growing source of introduction of non-native species worldwide [15]. Of all the parrots imported into China, some species (e.g., Monk Parakeet) are known to be highly invasive and have become invasive alien species in other countries or regions [6,22]. International trade involving these highly invasive species is expected to continue, making international trade a potential issue for the conservation of native biodiversity in many importing countries and regions, including China [10,53]. There have been no reports or studies of highly invasive parrots such as Monk Parakeets becoming invasive species in China, but more attention needs to be paid to these potential threats, and there is a need for prevention, monitoring, early warning, and ecological risk assessment of biological invasion before importing these species, given that Monk Parakeet is one of the major parrots imported to China.

The international parrot trade recorded in the CITES Trade Database is legal and just for CITES-listed parrots; it is thus only a small fraction of the total parrot trade in the world. The amount of unrecorded illegal and domestic parrot trades that exist around the world is even more alarming, and the illegal and domestic trades are a much greater threat to parrot populations in the wild and need to be given much more attention. Therefore, close monitoring of domestic trade and illegal trade in parrots and more studies of their potential impacts on wild populations may be more important than studies relying on CITES databases [10,24,38].

## 5. Conclusions

This study suggests that China’s import and export of parrots account for about 5% of the legal international parrot trade, and the overall volume of China’s international trade in parrots, particularly the export quantity, has been declining in the past 20 years. China’s international parrot trade routes have changed with time, with the major destination of its parrot exports shifting from Europe to Africa and the Arabian region, while more parrots are being gradually imported from countries in South America and Africa. The vast majority of parrots exported from China are from captive breeding, while a substantial proportion of parrots imported to China are wild-sourced and the proportion increased in recent decade. More attention needs to be paid to monitoring and control of China’s import of increasing proportion of wild-sourced parrots from some South American countries. Existing evidence shows there are a large-scale parrot captive breeding industry and extensive domestic parrot trades in China, which is an essential piece of the global picture of parrot trade warranting close monitoring and more studies in order to prevent captive breeding from being used to launder wild-caught parrots. Meanwhile, wildlife enforcement needs to be strengthened to crack down on illegal parrot (wildlife) trade.

## Figures and Tables

**Table 1:** Main species and quantity of parrots China imported and exported (1981–2022).

Imported Species	Quantity	Exported Species	Quantity
Grey Parrot *Psittacus erithacus*	21,201	Fischer’s Lovebird *Agapornis fischeri*	286,230
Monk Parakeet *Myiopsitta monachus*	12,627	Rosy-faced Lovebird *Agapornis roseicollis*	196,924
Yellow-collared Lovebird *Agapornis personatus*	9848	Yellow-collared Lovebird *Agapornis personatus*	117,669
Sun Conure *Aratinga solstitialis*	9099	Lord Derby’s Parakeet *Psittacula derbiana*	3137
Rosy-faced Lovebird *Agapornis roseicollis*	8880	Grey-headed Lovebird *Agapornis canus*	800
Orange-winged Amazon *Amazona amazonica*	7768	Senegal Parrot *Poicephalus senegalus*	300
Blue-and-yellow Macaw *Ara ararauna*	6943	Grey Parrot *Psittacus erithacus*	241
Red-and-green Macaw *Ara chloropterus*	6783	Rose-ringed Parakeet *Psittacula krameri*	207
Senegal Parrot *Poicephalus senegalus*	6449	Coconut Lorikeet *Trichoglossus haematodus*	165
Turquoise-fronted Amazon *Amazona aestiva*	5535	Orange-winged Amazon *Amazona amazonica*	151
Rose-ringed Parakeet *Psittacula krameri*	4680	Sordid Conure *Pyrrhura molinae*	100
Yellow-crowned Amazon *Amazona ochrocephala*	4561	Red-breasted Parakeet *Psittacula alexandri*	88
Fischer’s Lovebird *Agapornis fischeri*	4073	White Cockatoo *Cacatua alba*	57
Coconut Lorikeet *Trichoglossus haematodus*	3055	Red-and-green Macaw *Ara chloropterus*	53
Black-headed Parrot *Pionites melanocephalus*	2753	Yellow-crested Cockatoo *Cacatua sulphurea*	38

**Table 2:** Number of individual parrots China exported and imported for different purposes from 1981 to 2022.

Purpose	Import Quantity	Proportion (%)	Export Quantity	Proportion (%)
Breeding in captivity or artificial propagation (B)	8851	5.70	6	--
Educational (E)	20	0.01	--	--
Reintroduction or introduction to the wild (N)	174	0.11	--	--
Personal (P)	115	0.07	88	0.05
Circus or travelling exhibition (Q)	65	0.04	6	--
Scientific (S)	3	--	--	--
Commercial (T)	134,106	86.33	607,510	99.75
Zoo (Z)	7022	4.52	227	
Unknown	4983	3.21	1150	0.19
**Total**	**1** **55** **,** **339**	**100.00%**	**60** **8** **,** **9** **8** **6**	**100.00%**

**Table 3:** Number of individual parrots China exported and imported from different sources from 1981 to 2022.

Source	Import Quantity	Proportion (%)	Export Quantity	Proportion (%)
Captive-bred animals (C)	95,051	61.19	602,803	98.98
Animals born in captivity including F1 and subsequent (F)	3260	2.10	16	-
Confiscated or seized specimens (I)	292	0.19	10	-
Pre-convention specimens (O)	14	-	-	-
Specimens of animals reared in a controlled environment (R)	1	-	-	-
Source unknown which must be justified (U)	8	-	10	-
Specimens taken from the wild (W)	47,942	30.86	364	0.06
Unknown	8771	5.65	5783	0.95
**Total**	**1** **55,** **3** **39**	**100.00%**	**60** **8,986**	**100.00%**

**Table 4:** Main countries/regions exporting Grey Parrots to China.

Exporting Parties	Quantity	Wild-Caught	Others	Original Parties	Quantity
Congo (K)	2000	2000	0		
Congo (B)	620	620	0		
Spain	118	118	0	Cameroon	118
Mali	4476	1110	3336	Guinea	30
				Mali	1080
United Kingdom	40	40	0	Côte d’Ivoire	40
Malaysia	80	50	30	XX	30
				Malaysia	20
Mozambique	300	0	300		
Singapore	2638	1964	674	Congo (K)	1790
				Congo (B)	150
				Ghana	24
Thailand	30	20	10	Côte d’Ivoire	20
Netherland	25	20	5	Cameroon	20
South Africa	10,787	90	10,697	Congo (K)	40
				Congo (B)	50
**Total**	**21,114**	**603** **2**	**1** **5,** **0** **52**		**3412**

## Data Availability

All data analyzed in this article can be downloaded from the CITES Trade Database (available online: https://trade.cites.org (accessed on 6 August 2024)).
